# A Novel COVID-19 Diagnostic System Using Biosensor Incorporated Artificial Intelligence Technique

**DOI:** 10.3390/diagnostics13111886

**Published:** 2023-05-28

**Authors:** Md. Mottahir Alam, Md. Moddassir Alam, Hidayath Mirza, Nishat Sultana, Nazia Sultana, Amjad Ali Pasha, Asif Irshad Khan, Aasim Zafar, Mohammad Tauheed Ahmad

**Affiliations:** 1 Department of Electrical and Computer Engineering, Faculty of Engineering, King Abdulaziz, Jeddah 21589, Saudi Arabia; mohammad.mottahir@gmail.com; 2 Department of Health Information Management and Technology, College of Applied Medical Sciences, University of Hafr Al-Batin, Hafr Al-Batin 39524, Saudi Arabia; mmalam@uhb.edu.sa; 3 Department of Electrical Engineering, College of Engineering, Jazan University, P.O. Box 706, Jazan 45142, Saudi Arabia; 4 Department of Business Administration, Applied College, Jazan University, P.O. Box 706, Jazan 45142, Saudi Arabia; 5 Government Medical College Siddipet, Ensanpalli, Siddipet District, Telangana 502114, India; 6 Aerospace Engineering Department, King Abdulaziz University, Jeddah 21589, Saudi Arabia; 7 Computer Science Department, Faculty of Computing and Information Technology, King Abdulaziz University, Jeddah 21589, Saudi Arabia; 8 Department of Computer Science, Aligarh Muslim University, Aligarh 202002, India; 9 College of Medicine, King Khalid University, Abha 62217, Saudi Arabia

**Keywords:** biosensor, artificial intelligence, COVID-19, optimization, feature extraction, hyperparameter

## Abstract

COVID-19, continually developing and raising increasingly significant issues, has impacted human health and caused countless deaths. It is an infectious disease with a high incidence and mortality rate. The spread of the disease is also a significant threat to human health, especially in the developing world. This study suggests a method called shuffle shepherd optimization-based generalized deep convolutional fuzzy network (SSO-GDCFN) to diagnose the COVID-19 disease state, types, and recovered categories. The results show that the accuracy of the proposed method is as high as 99.99%; similarly, precision is 99.98%; sensitivity/recall is 100%; specificity is 95%; kappa is 0.965%; AUC is 0.88%; and MSE is less than 0.07% as well as 25 s. Moreover, the performance of the suggested method has been confirmed by comparison of the simulation results from the proposed approach with those from several traditional techniques. The experimental findings demonstrate strong performance and high accuracy for categorizing COVID-19 stages with minimal reclassifications over the conventional methods.

## 1. Introduction

Numerous dangerous viruses with zoonotic characteristics that can spread from animals to people make up the Coronaviridae family [1]. The risks and global issues posed by SARS-CoV-2/COVID-19 have created chances to use cutting-edge techniques for a healthy existence [2]. Due to its high contagiousness, this virus spreads more when people come into physical contact with infected persons. A COVID-19 strain of concern has been noted to be more contagious and more likely to result in breakouts or reinfections in those who have received vaccinations or previous infections [3]. These variations are more likely to result in serious illness, avoid detection by diagnostic tools, or resist antiviral therapy. The COVID-19 subtypes designated as variants of concern are Omicron, Alpha, Beta, Gamma, and Delta [4,5]. The rapid spread of these viral infections necessitated the development of low-cost diagnostic, therapeutic, and control strategies [6]. The ongoing challenges are the standard treatment of diseases in hospitals and the detection of epidemic infections in present clinicians [7]. Instead of sending samples to specialized research labs for RT-PCR decoding and diagnosis, specimens are sent out for molecular testing that is still not up to the quality of point-of-care (POC) diagnostics [8].

POC diagnostic kits that can operate without the aid of a technical expert and can also be used with various specimen types are needed for diagnosing pathogenic viruses. Scientists have created multiple technologies to detect infections in lab settings or use biosensors [9]. Antigen-based and molecular-based approaches are two frequently used methods. However, these methods have flaws, such as the time-consuming, careless virus isolation from cell culture that requires a high biosafety level and specialized knowledge [10]. Although the RT-PCR method requires a heat cycler and more advanced real-time PCR instruments, it is more sensitive than the antigen-based approach. The sensor can generate a wide range of data based on the inputs but the IoT bridges the gaps in equipment by delivering data quickly via a logical system [11]. The gradual miniaturization of electronic biosensors over the last few decades has allowed them to be incorporated into wearable devices, enabling continuous monitoring of physiological data [12]. The current study aims to evaluate the effects of different constant variants [13].

Artificial intelligence (AI) examines computer code for particular characteristics and creates high-performing algorithms [14,15]. Healthcare approaches based on AI perform better for broad-level diagnostics and control. The actual influence on the general health state of the population is comprehended, and the focus is on evaluating diseases’ regional variance, accessible healthcare technologies, and optimum preventive tactics [16,17]. Delivering a precise, quick, and cost-efficient prognostic model is necessary to significantly control the vaccine’s lack. Recently, disease diagnosis has benefited substantially from deep learning and optimization techniques [18,19,20]. However, it has not obtained the desired outcomes for COVID-19 variations. This is why a clever artificially intelligent-based strategy is created for the COVID-19 diagnosis with the functioning of human body systems.

This research’s main contribution can be summarized as follows: First, gather the COVID-19 data over a year using different datasets from innovative biosensor technologies. These datasets include patient symptoms, body condition, COVID-19 kinds (Alpha, Beta, Gamma, And Delta), and recovered cases. The purpose of the data preprocessing function is to remove undesired noise and data. Then, optimal selection and feature extraction are given using the principal component of African buffalo optimization (PCABO). Finally, a shuffle shepherd optimization-based generalized deep convolutional fuzzy network (SSO-GDCFN) is suggested to diagnose the COVID-19 disease state, types, and recovered categories. The suggested generalized deep convolutional fuzzy network combines the deep convolutional method with generalized approximation reasoning-based intelligent control. Here, the SSO algorithm is used to improve the method’s parameters. The MATLAB tool is used to carry out this research’s implementation. The effectiveness of the suggested strategy has been confirmed by a comparison of the simulation results from the proposed method with those from several traditional approaches.

The rest of this research article is articulated as follows. Section 2 explains the recent literature related to this work. The proposed methodology is detailed in Section 3. The result and performance comparative discussion is provided in Section 4. Finally, the article terminates with the conclusion of Section 5.

## 2. Related Work

The following is a summary of a few recent papers connected to this research. For creating a reliable sensor to detect COVID-19, Lim, Syazana Abdullah et al. [21] concentrated on the electrochemical performance of biosensors and AI technologies. However, the appropriate validation is needed for the successful investigation in this field. It is becoming clear that, to control the COVID-19 pandemic, the development of quick, focused, and responsive diagnostic methods for the preliminary phase of the SARS-CoV-2 virus, protein recognition is a critical response. These systems will produce the bioinformatics required for practical, innovative diagnostics, therapy optimization, and examination of therapies with better sensitivity.

As the best option to handle the pandemic, Kaushik, Ajeet Kumar et al. [22] advocated exploring the AI methodology by creating and constructing nanoenabled miniature electrochemical biosensors to recognize the SARS-CoV-2 virus at the location of the outbreak. An AI-powered collaborative biosensing technique generates the insights needed for preliminary phase COVID-19 diagnosis, viral load relationship with pathogenesis, understanding pandemic development, medication optimization, POC diagnoses, and disease monitoring in a tailored manner.

Due to its superior qualities, Paladhi, Amogha G. et al. [23] employed graphene-based electrochemical nano-biosensors. Fluorescence analysis is one of the exact methods of detecting optical biosignals that aid in acquiring real-time data with great precision and negligible fluctuations. Nevertheless, AI-assisted forecasts for the COVID-19 outbreak have been determined to be wrong or unreliable due to abundant extreme examples and chaotic social networks, enormous data arrogance, and algorithmic instability.

The difficulties with conventional technologies and recently developed biosensors are the COVID-19 nanotechnology-based detection kit and wearable-equipped nano-biosensors. Pradhan, Anchal et al. [24] discussed reverse transcription-loop-based mediated isothermal amplification (RT-LAMP) biosensors and the less well-known, but essential, piezoelectric nano-biosensor. However, effective performance metrics have not been used to confirm prior predictions of other illnesses.

The relevance of AI in improving the features of IoMT and point-of-care (POC) equipment used in sophisticated healthcare sectors, including cardiac monitoring, cancer detection, and diabetes management, is discussed by Manickam, Pandiaraj et al. [25]. This research also covers the technical and engineering difficulties and the potential of AI-based cloud-integrated individualized IoMT devices for developing effective POC healthcare systems appropriate for the future generation of intelligent healthcare.

Samples from COVID-19 patients are gathered and biomarkers were found utilizing various sensors with measurable indicators by V. Hemamalini and others [26]. The chest X-ray scans of the COVID-19 patients are then used to profile the interpolated data. An AI model called multilayer perceptron (MLP) is used to find the biomarkers’ secret signatures. Three parameters and a limit of detection are used, and the effectiveness of the biosensor is evaluated by creating calibration plots that closely match the model.

Umar Ibrahim, Abdullahi et al. [27] used artificial intelligence-driven techniques to investigate the 2019 new coronavirus pandemic, preceding epidemics, diagnostics, therapies, and medication detection based on categorization and prediction. They also discussed onsite and in-lab molecular testing utilizing CRISPR-based biosensing techniques. However, the drawbacks of laboratory methods and open-research problems in CRISPR-based biomedical applications and AI for treating Coronaviruses are currently being discussed. In addition, however, some biosensors need an amplification step, which lengthens the processing time.

Wearable biosensors may be used to continually monitor a variety of physiological markers for the early identification of COVID-19 clinical progression, according to research by Wong, Chun Ka et al. [28]. A smartphone app termed Biovitals Sentinel will receive physiological information in real time to identify minute physiological changes; this data will be analyzed utilizing a cloud-based multivariate physiology automation tool. The time it took to diagnose COVID-19 is the main result. However, the single-centeredness and exploratory nature of the clinical experiment has its limitations. The summarization of the literature review is detailed in Table 1.

## 3. Proposed Methodology

A powerful tool for using massive data models is AI-based diagnostics. In the proposed study, the COVID-19 data are gathered and grouped according to their demographic characteristics into categories of various variants. The biosensors used for the detection include blood pressure, G-FET-based, electrochemical, and potentiometric sensors. Following data preprocessing, the features are extracted using the PCABO method. To recognize and identify the COVID-19 variations utilizing an SSO-GDCFN approach, the suggested model also uses AI methods.

**Figure 1:**
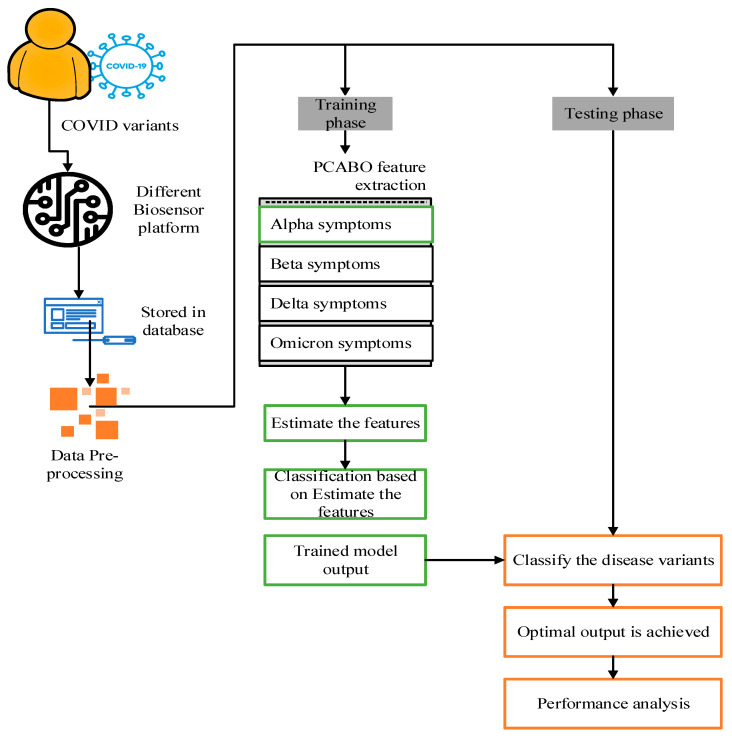
Proposed model’s architecture.

The Kaggle dataset’s multiclass probes are categorized into COVID-19 positive/negative and other variations, including Alpha, Beta, Delta, and Omicron, using AI-based screening to detect hidden characteristics during COVID-19 testing. Figure 1 depicts the suggested model’s architecture. Both feature extraction and classification are employed in the two AI steps. Finally, AI-based evaluation techniques offer superior outcomes in identifying the many COVID-19 viral variants.

### 3.1. Biosensor Technology

Biosensors are essential for understanding how molecules interact within the human body. This sensor can provide different outputs with varying ion sensitivity depending on the charge-transfer method. In this work, the COVID-19 samples are obtained using a variety of biosensors, including blood pressure sensors, G-FET-based sensors, electrochemical sensors, and potentiometric sensors, by determining an array of biomarkers [26]. Infection with COVID-19 is frequently brought on by hypertension. A flexible patch-like device called a blood-pressure sensor uses ultrasonic pulses to detect blood pressure. In the proposed study, G-FET-based sensors serve as a detection platform for COVID-19 spike antibodies, and the sensor recognizes the target antigen protein. This sensor demonstrated how the COVID-19 spike antibody could be more effectively synthesized than a graphene sheet, resulting in more accurate specimen identification. The electrochemical biosensors’ enzymes start the process and boost electron transport to the electrode’s surface. The reference electrode receives these electrical impulses, after which the output current is measured. The applied voltage in the blood sample solution triggers the electrochemical reaction. Finally, redox is output by potentiometry. The bioperception elements are coupled into potentiometric sensors, where the electrodes identify the catalysts. The COVID-19 patients’ swabs are placed in the sensor plates, which serve as the analytic.

### 3.2. Dataset Details

Data is gathered daily from Our World and published to the data GitHub repository for COVID-19. The data is about COVID-19 variations. The COVID-19 variations data include where the variants information is supplied, the date of data input, the variant, and the % of variants from the overall sequence number. Alpha, Beta, Gamma, Delta, Omicron (BA.1, BA.2, BA.4, BA.5, BA.2.12.1, BA.2.75, BQ.1, and XBB), and recombinant others are among the variants that were taken into consideration for validation.

### 3.3. Data PreProcessing

A valid data sheet was created as a first step based on the data’s accessibility. Converting unstructured data into an understandable format increases the classifier’s efficacy. After that, remove any extraneous or blank values from the current list. In this study’s COVID-19 global variant dataset, many low or null values have been supplied using the imputation method. The basic premise of the simple imputation strategy is to attribute the mean of the expected data or the nominal characteristic with the most significant predicted values instead of each missing value.

### 3.4. PCABO-Based Feature Extraction

The preprocessing work has already been completed to improve the features that are extracted for classification in the future. We extract specific characteristics by combining the principal component analysis with an African buffalo optimization. By integrating the PCABO technique, the performance of effective-feature vector extraction is attained. The algorithm may then be used to extract features from the raw data to prepare it for SSO-GDCFN model training. In our investigation, combining these two techniques can significantly influence the variables’ importance and extraction. By transforming an extensive collection of variables into a reduced one that still includes most of the data in the more comprehensive set, the PCABO dimensionality-reduction approach is frequently used to decrease the dimensionality of massive data sets. The technique reduces a data set’s number of variables while retaining as much data as feasible. First, normalize the dataset to ensure that all the continuous starting variables are equally important to the study. Then, for each variable value, standardization is performed by deducting the mean and dividing by the standard deviation using Appendix A. After standardization is complete, all the variables are scaled to the same value. This stage aims to comprehend that each variable’s departure from the mean relates to the various variables in the input data set. Since variables can occasionally be highly connected to the point where they include duplicated data. We construct the covariance matrix to find these associations per Appendix A. To identify the primary components of the data, we must calculate the linear algebra notions of eigenvectors and eigenvalues from the covariance matrix, as explained in Appendix A.

Select the principal component using African buffalo optimization (ABO) and develop the feature vector. Then, initialize the algorithm parameter and eigenvalues in optimization. The ABO is an innovative, naturally inspired algorithm that mainly works on three features improved communication, memory capacity, and extraordinary intelligent performance, and it is detailed in Appendix A. The recommended study’s objective is to gather more valuable characteristics that are perfect for SSO-GDCFN input to boost accuracy.

### 3.5. SSO-GDCFN-Based Classification

**Figure 2:**
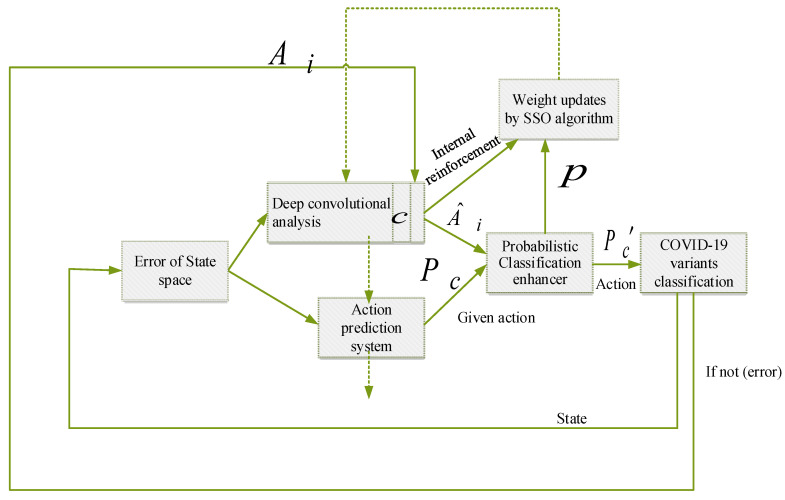
Proposed HO-GINFC predictive control methodology.

The SSO-GDCFN combines optimization and hybridization of deep learning and a fuzzy approach. The SSO algorithm optimizes the parameters of GDCFN. The architecture of the proposed SSO-GDCFN method is illustrated in Figure 2.

It comprises a stochastic action modifier, an optimal control system, and an action state evaluation system. The feed-forward action state evaluation system of GARIC has five tiers.

The optimal control system uses fuzzy inference *F_c_* to translate a state vector into a suggested action. The action–evaluation system maps a malfunction signal and controller parameters into a scalar score representing the state’s quality. The probabilistic movement enhancer uses both *F_c_* and *R_i_* to create an activity $\left(F_{c}^{\prime }\right)$ performed in the cold thermal storage-based air-conditioning system. In addition, this is utilized to provide internal reinforcement *R_i_*. The resultant state and a Boolean malfunction signal are delivered and returned to the controller. The two networks’ basic parameters are tweaked to create learning; the action–evaluation system changes the weights. The parameters representing the fuzzy membership functions vary during the robust control. A detailed study of this proposed methodology for COVID-19 variant classification is provided below:

(a) GDCFN approach:

The GDCFN approach is the improved neuro-fuzzy method. The deep convolutional approach improves the neural network of this system. In the GDCFN process, three main steps are categorized: deep action analysis, action chosen model, and probabilistic classification enhancer. The deep convolutional system replaces the current action–evaluation analysis.

***Deep convolutional analysis:*** The input layer of the deep action analysis is initially given the feature vector produced by the COVID-19 versions of the patients. Additionally, convolutional filters are used to create feature maps. Layer-to-layer connections between neurons are made possible by varying loads. The output estimation is detailed in Appendix B. Furthermore, the batch normalization function is applied. This layer is set up between the convolutional layer and the ReLU layer to improve the training properties of the suggested classification model. Significant training is achieved in this layer by controlling the network’s gradients and activations. Finally, the nonparametric ReLU layer, free of bias and weight, transmits the feature maps from the convolutional layers.

Furthermore, the max-pooling function is executed. The feature maps produced from the convolutional layers in the max-pooling layer are downscaled to decrease the data and the spatial size. By training the classifier, the proposed SSO strategy allows for the most accurate evaluation of the deep convolutional analysis weights. The FC layer’s output is regularized using the SoftMax activation function to process the classification layer effectively. The deep evaluation estimation layer, which makes use of the possibilities created by the SoftMax activation function to all the inputs to assign a class for each input data, is the last layer of deep convolutional.

***Action prediction system:*** This system selects a course of action based on the featured state and an inference strategy based on fuzzy-control concepts. Each layer of the system, which has five total layers, is responsible for a certain level of fuzzy inference. The input layer, which is the top layer, comprises input parameters with actual values. They may also be known as parameter estimations in linguistics. These nodes do no computations. The node computation $\zeta_{high}(x)$ goes into layer two if one of the fuzzy words in layer 1 has a high potential, such as when big is one of the output permutations for x. The ‘if’ component of any rule that uses it will significantly impact the output, and there will only be one input, explained in Appendix B. The spreads’ specification of length scales on each side of the center makes asymmetry conceivable.

The distributions provide length levels on each side of the mean, allowing for asymmetry, and the mean *f_a_* acts as a reference point. If required, further criteria are added in Appendix B. The center and dispersion are thought of as weights on the input connections. At layer 3, one rule must incorporate each of the mentioned conditions. A rule in the fuzzy rules correlates to a node in layer 3. Every layer 2 node involved in the IF portion of the rule sends data to it (Appendix B). The label that follows refers to a layer 4 node. It collects comments for all rules that use this specific consequent label. This node chooses the specific output action for each of the inputs $K_{A_{i}}$ in accordance with the rule $A_{i}$, (Appendix B). Since the pooling of subsequent labels is permitted, which is a distinguishing feature, a unit in layer four can have several outputs expressing various values. Every rule must have a corresponding feature action, giving it a degree that is then transferred to the next layer (Appendix B).

Despite the numerous inputs, the outcome is adequate. Due to the obvious way that the computation is done in the layer below, this adjustment is easy. Layer 5 will have an equal number of nodes as output action variables. The recommendations from each output node in the rule base are combined using the weighted sum shown below, where the weights stand for the robustness of the fuzzy control rules.

(1) $$P_{c}=\frac{\sum_{A_{i}=1}^{}K_{A_{i}}\beta_{}^{-1}(K_{A_{i}})\;}{\sum_{A_{i}=1}^{}K_{A_{i}}}$$

where *P* is always formed if the antecedent label functions surround every dimension of the input space. Layers 2 and 4’s input connections are the only ones with adjustable weights. The remaining weights are maintained at one. This shows that the gradient descent algorithm only employs two of the five layers of weights.

***Probabilistic Classification enhancer:*** This stochastically generates $P_{c}^{\prime }$ an action, a Gaussian random parameter with mean $P_{c}$ and standard deviation $\sigma \left({\hat{A}}_{i}\left(t+1\right)\right)$, using the values from the previous iteration ${\hat{A}}_{i}$ and the step $P_{c}$ suggested by the action control function. This is a non-negative, uniformly degrading, actual function that is actually used (for instance, the COVID-19-variant classification). This function exists $\sigma \left(\right)$ and evenly degrades $\exp\left({\hat{A}}_{i}\right)$. The COVID-19-variant classification system is really the one to which the activity $P_{c}^{\prime }$ is being applied. The probabilistic interruption of the suggested action enables enhanced-condition structure exploration and generalization abilities. When ${\hat{A}}_{i}$ is low, the magnitude of the deviation $\left|P_{c}^{\prime }-P_{c}\right|$ is enormous, and when ${\hat{A}}_{i}$ is high, it is relatively modest $\left|P_{c}^{\prime }-P_{c}\right|$. When the last step was successful, the controller adheres to the fuzzy control principles; nevertheless, when the previous action failed, the controller deviates significantly from the proposal in a random manner. The output variable’s measurements and range of fluctuation should be considered while determining the function’s exact form $\sigma \left(\right)$, particularly its size and rate of reduction. Each sample interval’s signal indicates the disturbance.

(2) $$p(t)=\frac{\left|\left|P_{c}^{\prime }(t)-P_{c}(t)\right|\right|}{\sigma ({\hat{A}}_{i}(t-1))}$$

This is merely the action control function’s normalized departure from the suggested action. The control action will use this as a teaching tool. Finally, the output action achieved the proper classification of the COVID-19 variants.

**Figure 3:**
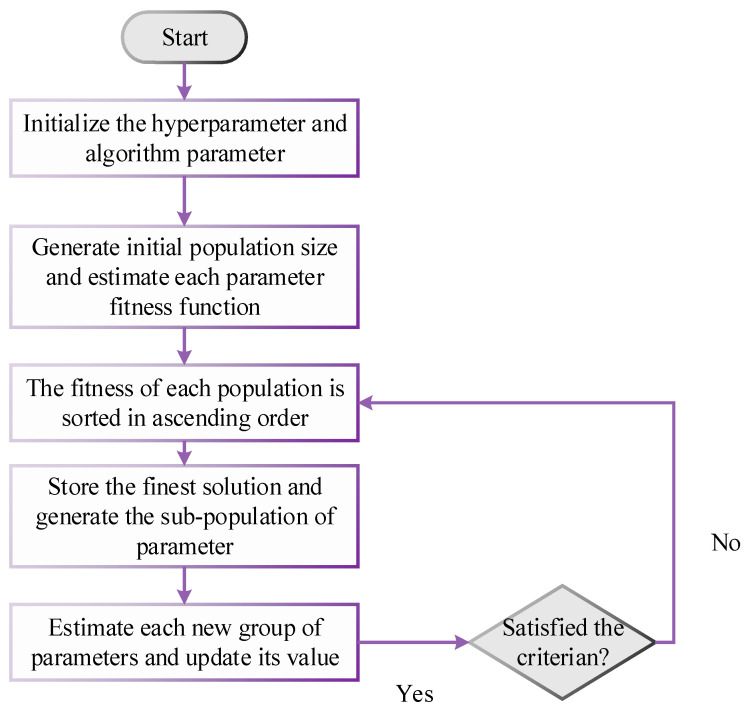
Flowchart of SSO algorithm.

(b) SSO for hyperparameter:

To trust the animals’ innate instincts to choose the best path to pasture, shepherds would put horses or any other animals together in a herd. For this purpose, the shepherd leads the sheep toward the horses. This conduct led to the creation of the SSO. The metaheuristic algorithm of SSO optimizes and enhances the learning parameter of the GDCFN approach (Appendix C). The flowchart of the SSO algorithm is illustrated in Figure 3.

## 4. Result and Discussion

The research cited in this section attests to the proposed method’s efficacy. The numerical and restricting programming language MATLAB 2018 Rb, developed by Math Works, implements the suggested approach. It operates on a Windows 7, 64-bit platform with an Intel 5 CPU and 4 GB RAM. In this experiment, all prediction models are trained on the training dataset, and their performance is evaluated on the testing dataset. The model incorporated the best characteristics before training and testing the prediction.

### 4.1. Evaluation Measures

We used several assessment and error metrics to choose the model with the most significant predictive power, including accuracy, precision, specificity, sensitivity/recall, the area under the curve (AUC), kappa, ROC, and mean square error (MSE).

***Accuracy:*** The most used performance metric for forecasts is accuracy. It is expressed as a percentage and indicates the proportion of correctly predicted occurrences. The predicted performance should be at or above 100% for better prediction results.

(3) $$\mathrm{Accuracy}\;\;=\;\;\;\frac{\;\vec{T}\vec{P}+\;\vec{T}\vec{N}}{\;\vec{T}\vec{P}+\;\vec{T}\vec{N}+\;\vec{F}\vec{P}+\;\vec{F}\vec{N}}$$

where, $\;\vec{T}\vec{P}$ is the number of correctly estimated variants and $\;\vec{T}\vec{N}$ is the number of correctly negative estimated variants.

***Precision:*** It is used to determine the total number of COVID-19-variants affected cases using the COVID-19 global dataset.

(4) $$\mathrm{Precision}=\frac{\;\vec{T}\vec{P}}{\;\vec{T}\vec{P}+\;\vec{F}\vec{P}}$$

***Sensitivity/Recall***: After accounting for false-negative and accurate-positive results, it is the proportion of positive samples.

(5) $$\mathrm{Sensitivity}/\mathrm{Recall}\;=\;\frac{\;\;\vec{T}\vec{P}}{\;\vec{F}\vec{N}+\;\vec{T}\vec{P}\;\;\;}$$

***Specificity:*** It is the proportion of models that actually made positive findings to all samples that produced positive results. Specificity is the proportion of true negatives to the sum of true negatives and false positives. For specificity, 1.0 has the highest value, while 0.0 has the lowest.

(6) $$\mathrm{Specificity}\;=\;\frac{\;\vec{T}\vec{N}}{\;\;\vec{F}\vec{P}+\;\vec{T}\vec{N}}$$

***Kappa:*** It is possible to calculate Cohen’s kappa using the following formula:

(7) $$k=\;\frac{P(i)-P(e)}{\;1-P(e)}$$

where, P(e) denotes probability coincidence and P(i) is the actual observed matching. ***Receiver Operating Characteristic (ROC):*** The percentage of true positives (sensitivity) vs. the rate of false positives (100-specificity) at various cutoff values of a parameter is plotted on an ROC curve. The sensitivity/specificity pair associated with each position on the ROC curve corresponds to a specific decision threshold.

***The Area under Curve (AUC):*** The AUC is one of the most often used metrics in the setting of imbalanced class populations, which will be used to evaluate performance in this experiment phase. The AUC metric defined by a specific point on the ROC curve for a binary issue is also known as balancing performance.

(8) $$\mathrm{AUC}=\frac{\left(\mathrm{Sensitivity}\;\mathrm{or}\;\mathrm{Recall}\right)+\mathrm{Specificity}}{2}$$

***Mean Squared Error (MSE):*** The *MSE* is a statistic used to assess how closely a fitted line resembles the sample points or the total of the squared deviations between the actual and predicted values.

(9) $$MSE=\frac{1}{m}{\sum_{i=A_{v},{\hat{P}}_{v}}^{m}\left(A_{v}-{\hat{P}}_{v}\right)}^{2}$$

where $A_{v}$ is the actual estimated variant, ${\hat{P}}_{v}$ is the predicted variant, and m is the number of global datasets.

### 4.2. Experimental Analysis

In the suggested paradigm, several biosensors are used to gather COVID-19 data from people, which are then fed appropriately into the biosensing platforms to analyze the bioperception components. Table 2 provides information on the biosensors employed in the system. First, the biosensing elements are made with the extracted materials. Following that, selected samples are chosen for additional diagnosis. Next, the detection limit is calculated for the COVID-19 viral testing and verifying related to the different biosensors and biosensing elements (Table 2). Finally, each sample is tested for the biosensors’ limit of detection.

These COVID-19 datasets were divided into testing and training datasets using the Kaggle dataset. Multiple types of COVID-19 variations are distinguished using the SSO-GDCFN classification algorithm. The COVID-19 viruses impact numerous COVID-19 variations, including Alpha, Beta, Delta, and Omicron. Patients with COVID-19 variants from various countries make up the dataset. Following sample synthesis, proposed AI algorithms are used to assess the biosensors for the analytical techniques. In the beginning, the target analysis is used to evaluate the selectivity and sensitivity of the sensors. Each day, information from Our World was collected and released to the COVID-19 data kaggle repository. The 100,417 COVID-19 samples are totally collected for examination. Consider that the number of samples is *x*. The input sample images are preprocessed for removing unwanted noise and errors. Moreover, 30% of those samples are utilized for testing, while 70% are used for training. Consequently, PCABO is used to find COVID-19’s best characteristics for each patient. The two-learning parameter value of ABO is 0.005. The important features are given to the SSO-GDCFN algorithm for disease classification. The experimental parameter detail for SSO-GDCFN is provided in Table 3.

Since the proposed approach SSO-GDCFN classification enables numerous classifications, it is used to improve clinical trials for drugs. For instance, if the person has high antispike protein as ≥200 IU/mL, antibody > 0–199 IU/mL, blood pressure > 1 Pa, and peak protein >/= 0.80 U/mL. Then, the proposed SSO-GDCFN validates the features state and classifies the COVID-19 variants as Delta. If the criteria are met, the classification continues till the final iteration. Consequently, the COVID-19 samples were classified using AI-based algorithms into the following categories: Alpha, Beta, Gamma, Delta, Omicron (BA.1, BA.2, BA.4, BA.5, BA.2.12.1, BA.2.75, BQ.1, and XBB), and Recombinant Others.

The COVID-19 samples were classified using AI-based algorithms into the following categories: Alpha, Beta, Gamma, Delta, and Omicron (BA.1, BA.2, BA.4, BA.5, BA.2.12.1, BA.2.75, BQ.1, and XBB), and Recombinant Others.

### 4.3. Performance Comparative Analysis

**Figure 4:**
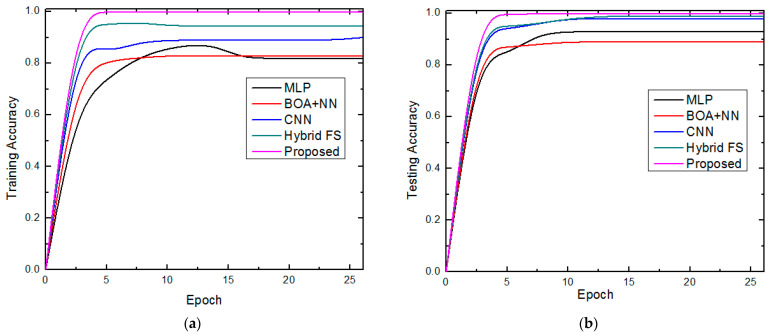
Comparison of the accuracy of (**a**) training and (**b**) testing.

In terms of accuracy, specificity, sensitivity/recall, precision, kappa, ROC, AUC, and MSE, the investigational analysis of the proposed methods for the data analytics of COVID-19 variants estimation outcomes is compared with the traditional methods such as MLP [26], BOA + NN [29], CNN [30], and hybrid FS [31]. Ref. [26] proposed the MLP method with biomedical sensor data for COVID-19 prediction. Ref. [28] introduced a neural network and improved the performance by a butterfly optimization algorithm based on the biomedical data for COVID-19 pattern estimation. The CNN model is studied by Ref. [29] for COVID-19 disease estimation based on the X-ray image dataset. Furthermore, the hybrid fully connected deep features with a forward-search algorithm and random-forest method are introduced in ref. [30]. The proposed method’s performance in terms of the training and testing accuracy is compared to that of traditional approaches in Figure 4. As demonstrated in Figure 4a,b, which displays the results of the testing accuracy, all systems perform well when utilizing an independent dataset for testing and in line with their performance during the training phase.

**Figure 5:**
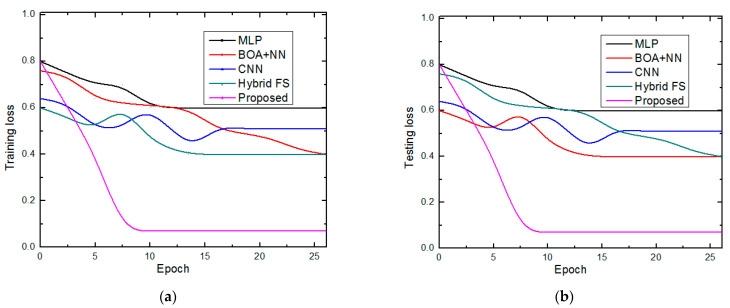
Comparison of the loss (**a**) training and (**b**) testing.

The suggested technique was identified as the best optimized hybrid deep learning method after a comparison of the performance of the five models, with a training performance of 99.98% accuracy and overall testing accuracy of 99.95%. Thus, the training accuracy of the suggested model was nearly 99.98%, which is extremely impressive. Since a small amount of information is lost during the training period, our hybrid approach will learn more quickly. The data are used to assess how effectively our model generalizes. Our model has the most-significant test accuracy when compared to other models such as MLP [26], BOA + NN [29], CNN [30], and hybrid FS [31]. We also use the SSO-GDCFN method to alleviate the over-fitting problem. The training and testing loss achieved by the proposed approach is compared to the traditional methods, as shown in Figure 5a,b.

**Figure 6:**
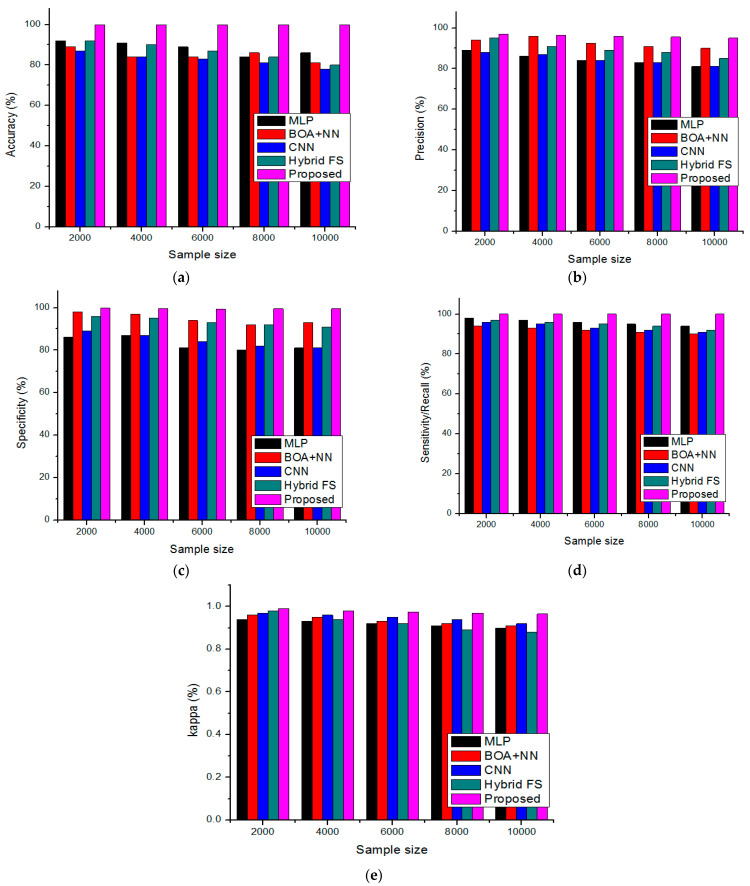
Comparison of performance metrics (**a**) accuracy, (**b**) precision, (**c**) sensitivity, (**d**) specificity, and (**e**) kappa.

Lower training loss facilitates better classification by anticipating the relationship between input and output labels. The training loss is calculated as a moving average of all processed batches. Since the loss is more significant in the early training phase when it declines quickly, the first batch of an epoch will have a significantly higher loss than the prior one. The results of the training loss will not reflect the loss that occurred at the end of the epoch but rather the average training loss that happened from the start to the end of the period. Our model also performed well on the validation set, as seen by the recommended model’s lower validation loss than other models. The proposed method consequences are compared with the earlier MLP [26], BOA + NN [29], CNN [30], and hybrid FS [31] are detailed in Figure 6a–e.

Moreover, the accurate estimation of COVID-19 variants is analyzed by the accuracy of the metric. The proposed method’s accurate results are compared with the existing techniques that are illustrated 99.98% for a 10,000-sample size and 99.9% for a 2000 for less sample size. This validation demonstrates that the accuracy values tend to decrease while increasing the sample size.

Furthermore, the proposed method’s precision-value results are compared with the existing methods illustrated in Figure 6b. The analysis shows that the proposed method has achieved higher precision, 99.9% for a 10,000-sample size and 99.2% for a 2000 for less sample size. This validation demonstrates that while increasing the sample size, the precision values tend to be less than the existing method; the proposed method’s precision value is high. The specificity/sensitivity value of the proposed method is compared with the current techniques, illustrated in Figure 6c,d. The sensitivity validation and recall metrics values are always identical. The values show that the proposed method specificity has achieved 95% for a 10,000-sample size and 97% for a 2000 for less sample size, along with 100% for both higher and lower data sizes. This validation demonstrates that while increasing the sample size, the precision values tend to be less compared to the existing method; the proposed method’s sensitivity and specificity value is high. The proposed strategy’s kappa value was determined as 0.965% for a sample size of 10,000 and 0.99% for a sample size of 2000 data. This validation shows that the proposed method’s kappa value is high, but the present methods tend to decrease when sample sizes are increased.

**Figure 7:**
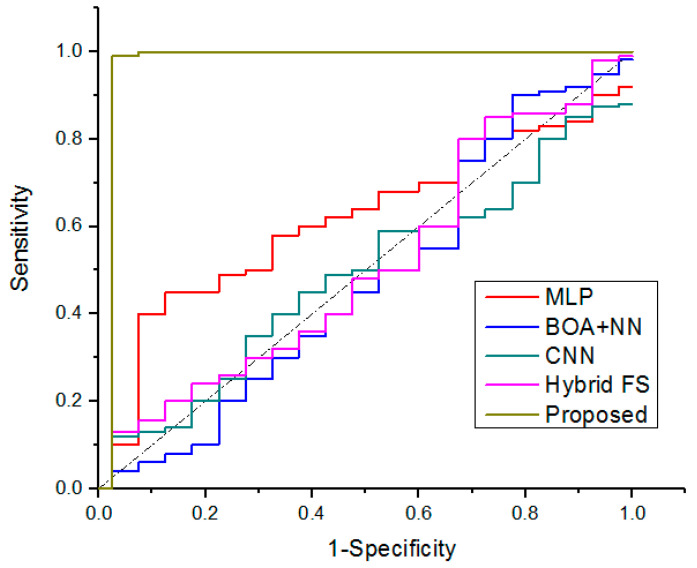
Comparative analysis of ROC curve for COVID-19 variant classification.

The 95% confidence interval of accuracy, sensitivity, and specificity on overall used approaches are analyzed by Berkman, N.D.’s [32] study. For each of the COVID-19 datasets, this study developed the receiver operating characteristic (ROC) curves that are shown in Figure 7. The ROC curve of a test with perfect discrimination (no overlap between the two distributions) passes through the upper left corner (100% specificity, 100% sensitivity). Consequently, the test’s accuracy increases as the ROC curve approaches its top left corner. The AUC value of the proposed approach is 0.98%, which is higher than the overall existing methods. The proposed SSO-GDCFN model has an AUC of 0.98 (95% CI 0.95–1.0), while the traditional approaches include MLP (0.92 [95% CI 0.1–0.92], BOA + NN (0.95 [95% CI 0.04–0.982], CNN (0.874 [95% CI 0.12–0.88]), and Hybrid FS (0.88 [95% CI 0.13–0.99]). The Delong test showed that the conventional model had a significant difference (significantly lower AUC) (*p* >0.05) when compared with the proposed technique (*p* < 0.05). The proposed scheme had the best sensitivity (1.00) but the lowest specificity (0.95) in terms of analytic metrics; other models provided the opposite findings, i.e., poor sensitivity (0.1–0.94) and specificity (0.8–0.9).

**Figure 8:**
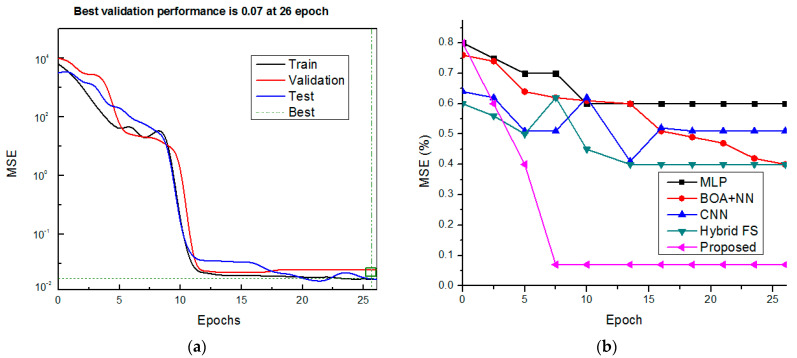
MSE metrics (**a**) proposed model output and (**b**) comparative analysis.

**Figure 9:**
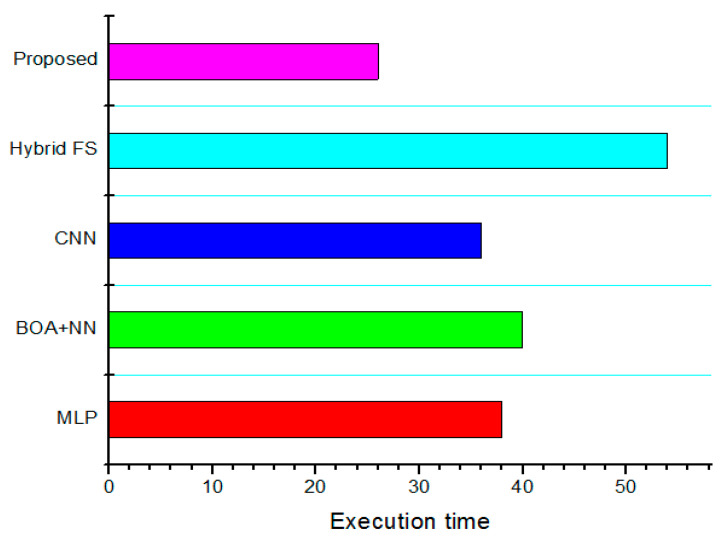
Comparative performance of execution time.

The MSE value of the proposed classification system and comparative analysis of MSE metrics are shown in Figure 8. The result demonstrates that the proposed method has achieved many ways, including MLP [26], BOA + NN [29], CNN [30], and hybrid FS [31]. Comparing the suggested technique to the existing MLP (38 s), BOA + NN (the 40 s), CNN (36 s), and hybrid FS (54 s) approaches, the processing time reduction is 26 s. Further, Figure 9 demonstrates the comparative study of execution time for the proposed method with other existing methods.

### 4.4. Discussion

The human population’s COVID-19 continues to change, raising questions about the validity of diagnostic tests created with an ancestral lineage in mind. A strain will not be able to recognize new varieties. Previous research has shown no difference in the sensitivity of several RDT tests between the Alpha, Beta, Gamma, and Delta and the ancestral strain. However, Omicron has only been examined in few research studies. It is necessary to employ AI techniques to assess the many COVID-19 variants based on biosensors since such a pan-variant of sensitivity antigen test is essential for public health. The overall comparison of proposed methodology metrics over the existing method is detailed in Table 4.

The findings demonstrate that the chosen SSO-GDCFN algorithm is compared to conventional methods under various circumstances, including several COVID-19 versions. When comparing the analysis results, the recommended SSO-GDCFN algorithm outperformed the current approaches regarding positive values for all metrics. Ref. [26] developed the MLP method for multiclass classification using X-ray images to estimate the hidden signatures. However, employing biosensors in this pandemic crisis is a considerable obstacle due to the extensive testing, which includes several features to assess the biosensors’ sensitivity and specificity. Therefore, this makes the system of MLP in COVID-19-variants classification complex and expensive. In Ref. [29], the BOA + NN method using CT images is developed for the COVID-19 diagnosis. However, the training and test data in the used datasets must be increased further to improve accuracy. Finally, in Ref. [30], the CNN model is used to diagnose COVID-19 by X-ray images.

Given that the model was trained incrementally in this work utilizing a variety of datasets, it was verified that CNN models need a sufficient amount of picture data for effective and correct classification. Yet, they need powerful computers and extensive training. In ref. [31], the author developed a hybrid FS method for ECG records-based COVID-19 disease detection. The provided tool might eliminate the drawbacks of PCR tests, antigen tests, and chest imaging methods. Yet, the class imbalance issues should be handled more effectively.

In comparison to standard MLP [26], BOA + NN [29], CNN [30], and hybrid FS [31] models, which have an accuracy of 87%, 83%, 79%, and 82% correspondingly, the chosen scheme has a more significant accuracy (99.8%). Furthermore, it is discovered that for all diagnoses, the created model’s sensitivity, specificity, accuracy, and kappa are higher than the present model’s. The accuracy of the analysis process is used to assess each method’s effectiveness. Comparing the suggested technique to the current methods MLP [26], BOA + NN [29], CNN [30], and hybrid FS [31], it shows that the new approach has provided good performance metrics for COVID-19-variant classification. The proposed investigation used the SSO-GDCFN method with biosensors for intelligent COVID-19 diagnosis. The proposed method has high specificity and sensitivity. In addition, reagent use for calibration is minimal, making the system reaction time rapid. This innovative biosensors’ capacity to detect the target the viral antigen with different variants quickly, in real time, in remote areas, and with great sensitivity can eventually lead to an early diagnosis of COVID-19. Further, this is used for the random modified COVID-19 variants due to the usage of significant feature sensing by biosensors. In comparison to the existing technique, the proposed algorithm has produced good results with a low time complexity.

## 5. Conclusions

The COVID-19 epidemic is still spreading worldwide and has not yet been halted. At the same time, research into better ways to stop the spread is underway. The primary purpose of biomedical sensors is to increase the sensitivity of objective analyses with biomedical equipment. The suggested model uses three different kinds of biosensors, including potentiometric, blood pressure, G-FET-based, and electrochemical sensors. This article gathered and examined studies on the recently identified COVID-19 virus types, which are highly contagious and rapidly mutate. The SSO-GDCFN classifier finds the COVID-19 variants’ confidential data when categorizing viruses. For determining the phases of COVID-19-infected individuals, the likelihood has been examined from the feature set. The results show that the accuracy of the proposed approach is as high as 99.99%. In addition, precision is 99.98%, sensitivity/recall is 100%, specificity is 95%, kappa is 0.965%, AUC is 0.98%, and less MSE is 0.07%, as well as 25 s. The experimental findings demonstrate strong performance and high accuracy for categorizing COVID-19 stages with minimal reclassifications over the traditional methods. In the future, a detailed study of each virus variant will be studied using this improved classification algorithm.

## Figures and Tables

**Table 1:** The summarization of the literature review.

Reference	Years	Methodology	Advantages	Limitation
Lim, Syazana Abdullah et al. [21]	2020	Biosensor and AI	Reliable sensors are used to detect the system	Not proper performance validation is achieved
Kaushik, Ajeet Kumar et al. [22]	2020	Biosensing strategy powered by AI	A detailed study is established for the analysis	Lack of performance analysis such as accuracy and other parameters
Paladhi, Amogha G. et al. [23]	2022	Electrochemical nano-biosensors and AI	High robustness	Surplus extreme examples; disorderly social networks; big data hubris; algorithmic instability; and incorrect or untrustworthy are the primary problems.
Pradhan, Anchal et al. [24]	2021	RT-LAMP	An intelligent sensing approach is obtained.	Effective performance metrics have not been used to confirm prior predictions of other illnesses.
Manickam, Pandiaraj et al. [25]	2022	POC and AI	An effective healthcare system is achieved for a proper diagnosis.	The computational burden is high.
V. Hemamalini et al. [26]	2022	MLP	The efficacy of the biosensor is evaluated and obtained.	However, the training time is more.
Umar Ibrahim, Abdullahi et al. [27]	2021	CRISPR and AI	A detailed study of challenges is investigated.	Need an amplification step, and the processing time is more.
Wong, Chun Ka et al. [28]	2020	Biovitals	The early detection result is accurate.	The single-centeredness and exploratory nature of the clinical experiment

**Table 2:** Biomedical sensors for selected sample diagnosis.

Type of Biosensor	Bio-Observation Element	Extracted and Desired Specimen	Range of Detection
Potentiometric	Protein, Viral RNA	Saliva Sputum, swab (Anti-spike protein)	10^1^ cfu mL^−1^
G-FET	Antigen/Antibody	IgM, IgG (Antibody)	1 fg mL^−1^
Blood Pressure sensor	Blood Pressure	Sample of Blood	1 Pa
Electrochemical	Molecular separation of viral RNA	IgM, IgG (Peak Protein)	10 μg mL^−1^

**Table 3:** Parameters of SSO-GDCFN.

Parameters	Values
Optimizer	SSO
Learning rate	0.001
Epoch size	26
Batch size	27
Dropout	0.5
Fuzzy rule	13
Linguistic labels	23

**Table 4:** Overall comparisons of proposed methodology metrics over the existing method.

Metrics	MLP [26]	BOA + NN [29]	CNN [30]	Hybrid FS [31]	Proposed
Accuracy (%)	87	83	79	82	99.99
Precision (%)	86	81	78	80	99.98
Sensitivity/recall (%)	94	90	91	92	100
Specificity (%)	81	90	81.05	85	95
Kappa (%)	0.9	0.91	0.92	0.88	0.965
AUC (%)	0.82	0.75	0.86	0.91	0.98
MSE (%)	0.6	0.4	0.51	0.4	0.07
Computational time (s)	25	40	36	54	26

## Data Availability

All datasets are available at https://www.kaggle.com/datasets, accessed in January 2023.

## Appendix A

For each variable value, standardization is performed by deducting the mean as well as dividing by the standard deviation using Equation (A1),

(A1) $$x=\frac{V-m}{\sigma }$$

where *V* is the value, *m* is the mean, and *σ* is the standard deviation.

The covariance matrix, which includes entries for all potential pairings of the starting variables, is an *n* × *n* symmetric matrix.

(A2) $$H=Cov\{x\}=E\left\{\left(x-\beta \right){\left(x-\beta \right)}^{T}\right\}$$

Estimate the eigenvalue η*_i_* and the eigenvector $E_{1},E_{2},\ldots \ldots \ldots E_{ni}$ and the value for *G* covariance is j=1,2,3…n. The descending function is used to sort the eigenvalues using Equation (A3),

(A3) $$\left|\eta I-H\right|=0$$

Randomly initialize the buffaloes to the n. The finest fitness value of buffalo is updated by Equation (A4),

(A4) $$e_{i+1}=e_{i}+l{\hat{p}}_{1}(b_{h.\max}-v_{i})+l{\hat{p}}_{2}(b_{h.\max}^{\prime }-v_{i})$$

where $e_{i}$ and $v_{i}$ are the exploitation and exploration of the $i^{th}$ buffalo (i=1,2…..n), the two learning parameters are represented as $l{\hat{p}}_{1}$ and $l{\hat{p}}_{2}$, respectively. The best fitness value from the group is defined as $b_{h.\max}$ and the best fitness value from the individual is presented as $b_{h.\max}^{\prime }$. The location of group and individual eigenvalues are expressed using Equation (A5),

(A5) $$v_{i+1}=\frac{\left(v_{i}+e_{i}\right)}{\pm 0.5}$$

If the criteria are met, then the optimal eigenvalue is obtained otherwise; repeat the process for iteration. Three primary components make up Equation (A5), including the memory section $e_{i+1}$, which shows that the eigenvalues are knowledgeable that they have moved from their original places $e_{i}$ to new ones. This demonstrates their vast memory, a critical component of their nomadic existence. The second section represents the species’ ability to work together $l{\hat{p}}_{1}(b_{h.\max}-v_{i})$. The herds can follow the location of the top eigenvalue in each iteration because they have exceptional communication skills $l{\hat{p}}_{2}(b_{h.\max}^{\prime }-v_{i})$. The final term highlights the remarkable intellect of these creatures in this equation. They can compare their current position to their prior greatest productive location. As a result, they are more equipped to make judgments about their quest for answers. Equation (A5) moves the buffaloes to a different place in response to the effects of Equation (A4). To pick which primary components to preserve, create a feature vector using Equation (A6),

(A6) $$f_{i}=\sum_{n}^{a}\left|E_{ni}\right|$$

where $i^{th}$ entry of $E_{n},i=1,2,3\ldots ..a,\;n=1,2,3\ldots ..a$ is denoted as $E_{ni}$ and the absolute value of $e_{vj}$ is represented as $\left|E_{ni}\right|$. The $f_{i}$ is sorted as the descending order function.

## Appendix B

***Deep convolutional analysis:*** The output of these layers is stated mathematically as,

(A7) $$\left(s^{t+1}\right)=\left(s^{t}\right)+\sum_{i=0}^{n}\sum_{h=p}^{n}\left(w_{i}\right)*\left(b_{i}\right)$$

where ∗ denotes the convolutional operator and $\left(s^{t+1}\right)$ represents the convolutional layer’s fixed-feature map. Assume that the suggested SSO method is adjusted to provide the bias $\left(b_{i}\right)$ and $\left(w_{i}\right)$ weights of the convolutional layers that are best.

Based on the feature map of the convolutional layer, the fully connected layer generates the final output as follows:

(A8) $$c=\alpha \left(s^{t+1}\right)\;\;\;with\;\;\left(s^{t+1}\right)=\left(s^{t}\right)+\sum_{i=0}^{n}\sum_{h=p}^{n}\left(w_{i}\right)*\left(b_{i}\right)$$

***Action prediction system:*** The function is expressed using Equation (A9),

(A9) $$\zeta_{f_{a},d_{la},d_{ra}}(x)$$

where, $f_{a},d_{la},d_{ra}$ denotes the center, left, and right spreads of the label’s fuzzy membership values, respectively, and *a* signifies a value in linguistics (e.g., medium error, high error, low error, etc.).

Here is an illustration:

(A10) $$\zeta \left(x\right)=\frac{1}{1+{\left|\frac{x-f}{d}\right|}^{z}}$$

The control of the curvature mechanism is z where $d=d_{la},\mathrm{or}\;\;\;d_{ra}$, hence x<f or x≥f. Next, Equation (A11) is used to do the triangular MF,

(A11) $$\zeta_{f,d_{l},d_{r}}(x)=\left\{\begin{array}{cc} \begin{array}{c} 1-\left|x-f\right|/d_{{}_{r}}\\ 1-\left|x-f\right|/d_{{}_{l}}\\ 0 \end{array} & \begin{array}{c} i\in \left[f,f+d_{{}_{r}}\right]\\ i\in \left[f-d_{{}_{l}},f\right]\\ otherwise \end{array} \end{array}\right.$$

The node carries out the min operation, which we softened to the matching continuous, separate softmin action.

(A12) $$K_{A_{i}}=\frac{\sum_{i}\zeta_{i}e^{-h\zeta_{i}}}{\sum_{i}e^{-h\zeta_{i}}}$$

The symbol $\zeta_{i}$ indicates the level of compatibility between a fuzzy label that appears as a single occurrence of a rule forebear $A_{i}$ and the relevant input parameter. This softmin approach $K_{A_{i}}$ produces the degree of rule application. Since the parameter h→∞ determines how smooth the softmin operation is, when we change h to it, we restore the usual min operator. However, for finite h, the inputs in this instance gain a differentiable component, making computing gradients throughout the learning process much more manageable.

This mapping is stated in the following way:

(A13) $$\zeta_{f,d_{l},d_{r}}^{-1}(K_{A_{i}})$$

where $f,d_{l},d_{r}$ denotes the label’s fuzzy membership values.

Results of the local mean-of-maximum for triangular functions

(A14) $$\zeta_{fa,d_{l}a,d_{r}a}^{-1}(K_{A_{i}})=\left(f_{a}+\frac{1}{2}(d_{ra}-d_{la})\right)\left(\sum_{A_{i}}K_{A_{i}}\right)-\frac{1}{2}(d_{ra}-d_{la})\left(\sum_{A_{i}}K_{A_{i}}^{2}\right)$$

Depending on the number of inputs, a polynomial $\zeta^{-1}(x)\;$ in x usually only requires one output.

## Appendix C

The mathematical model uses a randomly selected beginning-population parameter in the solution space to initiate SSO:

(A15) $$\begin{array}{cc} H_{a,b}^{0}=H_{\min}+rand\times (H_{\max}-H_{\min}); & \;\;\;a=1,2,\ldots ,\;u\;\;\;\mathrm{and}\;\;b=1,2,\ldots ,\;v \end{array}$$

where $H_{\min}$ and $H_{\max}$ are the parameters of the model’s minimum and maximum, respectively; rand is a random variable generated between 0 and 1 for each constituent; v is the number of people in each group; and u is the total number of groups. This method uses Equation (A16) to determine the community’s total population.

(A16) $$vH_{p}=u\times v$$

According to their goal functions, the first u members of each population are inserted at random into the first column of the multicommunity conditions Equation (A17) in this parameter tuning operation. The subsequent u members are chosen in a manner identical to the first stage, and they are positioned arbitrarily in the section to create the second column of the multi-community parameter. This process is performed several v times to build the multicommunity matrix as follows:

(A17) $$H_{p}=\left[\begin{array}{cccccc} H_{1,1} & H_{1,2} & \cdots & H_{1,v} & \cdots & H_{1,v}\\ H_{2,1} & H_{2,2} & \cdots & H_{2,v} & \cdots & H_{2,v}\\ \vdots & \vdots & \vdots & \vdots & \vdots & \vdots \\ H_{a,1} & H_{a,2} & & H_{a,b} & & H_{a,v}\\ \vdots & \vdots & \vdots & \vdots & \vdots & \vdots \\ H_{u,1} & H_{u,2} & \cdots & H_{\begin{array}{l} u,b \end{array}} & \cdots & H_{u,v} \end{array}\right]$$

It is significant to notice that each row of the multicommunity parameter reflects a group’s members, with the top column of the multicommunity parameter reflecting the best members of each group. In addition, the individuals in the last segment represent the group’s weakest variable. Each group member receives a unique step size based on two characteristics. The first variable $D_{a,b}^{worst}$ shows the ability to go further into the problem space. The second variable $D_{a,b}^{best}$ indicates the capacity to look around previously visited probable solution space regions. The step sizes of mathematical expression are as follows:

(A18) $$D_{a,b}^{}=D_{a,b}^{worst}+D_{a,b}^{best}._{\;}a=1,2,\ldots ,\;u\;\;\mathrm{and}\;\;b=1,2,\ldots ,\;v$$

Estimating the worst and best function of step size for parameter tuning is possible using Equation (A17).

(A19) $$D_{a,b}^{worst}=\kappa \times rand_{1}\times (H_{a,worst}-H_{a,best})$$

(A20) $$D_{a,b}^{best}=\lambda \times rand_{2}\times (H_{a,best}-H_{a,b})$$

For the value of the objective function, $H_{a,best}$ and $H_{a,worst}$ are the better and worse parameters, whereas $H_{a,b}$, $rand_{1}$ and $rand_{2}$ are random parameters with each component formed between 0 and 1. It is important to note that the community’s initial parameter lacks a superior affiliate. Therefore, $D_{a,1}^{best}$ is equal to 0. Due to the $u^{th}$ group’s final parameters, $H_{a,v}$ does not have a parameter that is worse than it. Hence, it is also zero. Additionally, κ and λ are the factors that affect exploitation and exploration, respectively. These variables’ definitions are as follows:

(A21) $$\kappa =\kappa_{0}+(\kappa_{\max}-\kappa_{0})\times t$$

(A22) $$\lambda =\lambda_{{}_{0}}-\lambda_{{}_{0}}\times t;\;\;\;\;\;\;\;\;t=\frac{\mathrm{Iteration}\;\mathrm{rate}}{\mathrm{Total}\;\mathrm{value}\;\mathrm{of}\;\mathrm{iteration}}$$

There will be t increases and κ drops as the number of iterations rises λ. As a result, exploitation advances faster than the exploration. The new parameter of $H_{u,v}$ is calculated using Equation (A23), per the preceding step. The parameter is then to be adjusted if it $H_{u,v}$ is not worse than its initial objective function rate:

(A23) $$H_{new\;u,v}=H_{u,v}+D_{u,v}$$

After a maximum number of iterations, the optimization process is finished. If not, it returns to the first step and repeats the process.
